# Socioeconomic status, BMI, and brain development in children

**DOI:** 10.1038/s41398-022-01779-3

**Published:** 2022-01-24

**Authors:** Evan Dennis, Peter Manza, Nora D. Volkow

**Affiliations:** 1 National Institute on Alcohol Abuse and Alcoholism, National Institutes of Health, Bethesda, MD USA; 2 National Institute on Drug Abuse, National Institutes of Health, Bethesda, MD USA

**Keywords:** Learning and memory, Human behaviour

## Abstract

Low socioeconomic status (SES) in childhood is associated with deficits in executive function and changes in cortical morphology. Furthermore, rates of childhood obesity are greater among low SES children and childhood obesity is also associated with cortical alterations and impaired neurocognition, specifically in the domain of executive function. To investigate the influence of BMI on the relationships between SES and both neurocognition and brain morphology, we used data from the Adolescent Brain Cognitive Development (ABCD) study to construct multiple linear regression models and conduct mediation analyses. Overall, SES as measured by household income, highest level of parental education, and area deprivation, was associated with lower BMI, greater total and prefrontal cortical volume, and better performance on assessments of executive function. Mediation analysis indicated that BMI had a significant indirect effect on associations between area deprivation and both total and prefrontal cortical volumes. BMI also played a mediating role in the associations between area deprivation and composite neurocognitive scores, which were driven by performance on tasks of working memory and cognitive flexibility, but not cognitive control. These findings suggest that BMI should be considered in future studies investigating the relationship between low SES and poor neurodevelopmental outcomes.

## Introduction

Socioeconomic disadvantage among children predicts a series of negative outcomes in cognition and academic achievement [1–3]. Children from low socioeconomic backgrounds are predisposed to a number of negative health risks early on and later in life [4–6]. Particularly, rates of childhood obesity are prevalent among children from lower socioeconomic backgrounds in the United States and other industrialized countries where low socioeconomic status (SES) populations have access to energy-dense diets [7, 8]. There are several complex factors that could be driving this relationship. Higher parental education is associated with more participation in sports and consumption of fruits and vegetables, and less screen time and consumption of soft drinks and fast food among children [9]. Neighborhood factors have also been suggested to play a mechanistic role. Nutritional and physical characteristics of deprived areas, such as fast-food outlet density and crime rates, may facilitate excessive calorie intake and discourage physical activity among low SES youth [10–12]. Childhood and adolescent obesity is also associated with a number of deficits in brain structure and cognition. Together, these findings suggest that body weight is an important target for understanding neurodevelopmental outcomes in low socioeconomic youths. However, the neurodevelopmental consequences of elevated BMI in the context of low SES children are not well characterized.

Several studies have investigated the association between greater BMI and lower scores on tests of neurocognition, specifically in the domain of executive function [13–16]. Sub-domains of executive function, including working memory, cognitive flexibility, and cognitive control (or attention), may play a role in food-related behaviors and several theories regarding the directionality of this relationship have been proposed [17–19]. For example, Wu et al. [20]. found that obese children perform significantly worse on working memory tasks than their lean counterparts, and that working memory ability mediated the negative association between obesity and academic performance. Higher BMI has also been associated with lower cortical volumes and other morphological alterations relative to lean children, most notably in regions of the prefrontal cortex responsible for executive function [17, 18]. Differences in prefrontal metabolic activity in both resting state and during cognitive tasks have also been reported among obese individuals. Both brain structural and functional alterations are suggested to underlie the relationship between BMI and cognition [19, 21–24].

Similar to obesity, low SES has been associated with both structural and functional differences in the cortex as well as deficits in neurocognition in children [25–29]. For instance, Lawson et al. [27]. found that parental education predicted prefrontal cortical thickness, particularly in the right anterior cingulate gyrus and the left superior frontal gyrus. Low SES children perform significantly worse than higher-SES controls on neurocognitive tasks, with the most pronounced differences observed in executive functions, including working memory and cognitive control [30]. Several factors have been suggested to facilitate these relationships, such as linguistic exposure and chronic stress as measured by cortisol levels [31, 32].

A number of studies have leveraged the large sample sizes provided by the Adolescent Brain Cognitive Development (ABCD) study to explore the aforementioned relationships in adolescents. Investigators using this dataset have found greater BMI to be associated with lower prefrontal cortical thickness and lower scores on tasks of executive function, with morphological differences partially mediating the relationship between BMI and executive cognition [19, 24]. Similarly, ABCD studies have demonstrated that low SES, and most specifically neighborhood disadvantage, is related to worse neurocognitive scores and reduced structural measures in frontal regions. Cortical deficits once again played a mediating role in the relationship between the other two variables [29, 33, 34]. Lastly, ABCD studies have shown that neighborhood disadvantage and individual measures of SES are associated with alterations in both task-based and resting-state functional connectivity related to reward anticipation and cognitive function [35–37].

In sum, many studies have observed significant relationships between BMI, SES, brain structure, and cognitive function, but they often measure only two of these variables and have done so in relatively limited samples. Few studies have examined how BMI and SES interact regarding their effects on brain structure and cognition, which could help disentangle some of these complex interdependencies. Here, we take advantage of the ABCD dataset, and investigate a potential model where BMI influences the associations between childhood SES and neurocognitive development. We hypothesize that the relationships between low childhood SES and both cortical volume and cognitive performance are partially mediated/moderated by BMI. Such a relationship would highlight the importance of improving accessibility of healthy food options and opportunities for physical activity in low-income areas and would suggest that developmental outcomes in low SES demographics could be improved through interventions aimed at maintaining a healthy body weight.

## Methods

### Data source

This investigation used data from the Adolescent Brain Cognitive Development^SM^ (ABCD) Study (https://abcdstudy.org), held in the NIMH Data Archive (NDA). This is a large-scale, longitudinal study following over 11,000 9-10-year olds for 10 years throughout development. Participants were recruited through school systems associated with 21 data collection sites across the United States. The recruitment protocol was designed to result in a study sample that is representative of the sociodemographic variation of the US population [38]. Ethical review and approval of the research protocol have been previously described [39].

The current study uses data from ABCD release 2.0, containing baseline data for 11,876 children. Participants lacking data for any of the variables of interest were not included in the statistical analyses. Furthermore, only one individual from each family was selected at random to remove any possible effects of relatedness between subjects, resulting in a sample size of 7607 participants.

### Measures

#### BMI

The height and weight of each subject was measured up to three times and averaged. BMI was calculated by multiplying 703 by mean weight in pounds divided by mean height in inches squared.

#### Socioeconomic status

Measures used to characterize SES in this study included Area Deprivation Index (ADI), highest level of parental education (PE), and annual household income (HI). ADI is a measure of socioeconomic disadvantage at the neighborhood level. It is based on 17 metrics derived from Census data describing poverty, education, employment, and housing quality, with a higher score indicating greater neighborhood deprivation [40, 41]. The weighted mean ADI of each subject was calculated based on the time spent at each reported address of residence and the corresponding ADI score.

PE and HI are widely used measures of SES and are considered essential for all mental health research by the National Institute of Mental Health [42]. These were measured based on parental questionnaires included in the ABCD study’s demographic battery [43]. The original survey item assessing PE had 21 different response options which were re-coded into 5 levels (Less than High School, High School Grad/GED, Some College, Associate’s/Bachelor’s Degree, and Postgraduate Degree). If PE for both parents was reported, the highest value of the two was used. The original survey item assessing HI had nine different response options with varying increments of income between them. As such, household income could not reliably be implemented as a continuous variable and was instead re-coded into three levels (<50 K, 50-<100 K, ≥100 K), roughly reflecting lower, middle, and upper class. These transformations have been implemented in previous studies using the ABCD data set and were implemented here to simplify our statistical models as much as possible without losing nuance [44].

#### Physical activity

Physical activity was measured by self-report as the number of days in the week prior to the interview that each subject was physically active for a total of at least 60 minutes per day. This data was collected via questionnaire from the Youth Risk Behavior Survey as part of the ABCD Study’s baseline physical health battery [43].

#### Neurocognition

A detailed description of the ABCD baseline neurocognitive battery is described by Luciana et al. [45]. Among other assessments, the baseline battery included the NIH Toolbox, a collection of seven tasks assessing different domains of cognitive function [46]. For our analyses we selected the age-corrected composite score on the NIH Toolbox as well as the age-corrected scores on three of its executive function subtests: The Flanker Task (a measure of cognitive control/attention), the List Sorting Test (a measure of working memory), and the Dimensional Change Card Sort Task (a measure of cognitive flexibility).

#### Structural neuroimaging

A detailed description of the complete imaging procedures of the ABCD study is described by Casey et al. [47]. The scanning protocol was designed to be implemented with three different 3T scanners (Siemens, General Electric, and Philips), allowing for data harmonization across 21 different imaging sites.

For the current study, we used the structural MRI data acquired from 3D T1-weighted images with a 1 mm isotropic resolution. The 3D T1-weighted images were acquired while the participant watched a child-friendly movie. Centralized processing and analyses of MRI data were conducted by the ABCD Data Analysis and Informatics Center. Real-time motion detection and correction were utilized on General Electric and Siemens Scanners. Signal-to-Noise Ratio and head motion statistics were automatically calculated for quality control. For manual quality control, images were reviewed by trained technicians, and those deemed unacceptable due to artifacts were not included in the data set [48].

Cortical volumes were constructed using FreeSurfer version 5.3.0 and segmented according to the Desikan–Killiany atlas [49]. Total Cortical Volumes for the prefrontal cortex (our primary region of interest) and occipital cortex (a control region) were calculated by summing the volumes of the regions of interest included in these areas. We also used bilateral total cortical volume.

### Statistical analyses

For each of the following analyses, a two-tailed alpha set to 0.05 was used for all inferences and false discovery rate was used to adjust for multiple comparisons.

### Multiple linear regressions

To begin exploring the role of BMI in the association between SES and Neurocognition/Cortical Morphology, we first established the relationship between SES and BMI and the relationships between SES and our different measures of Neurocognition/Cortical Morphology. Specifically, these relationships are a precondition for traditional mediation analysis [50]. To do this we generated different sets of multiple linear regression models using the lm function in R version 4.0.2 [51]. Our eight response variables included BMI, Total Cortical Volume, Prefrontal Cortical Volume, Occipital Volume, Total Composite Neurocognitive Score, and scores on the three selected Toolbox subtests: The Flanker Task, the List Sorting Working Memory Test, and the Dimensional Change Card Sort Task. Independent variables of interest included our measures of SES (ADI, PE, and HI). Covariates included age, sex, race, ethnicity, physical activity, and total intracranial volume. These covariates were selected as age, sex, race, and ethnicity were shown to be significantly associated with BMI, and it is important to control for intracranial volume in imaging studies [52–54]. Furthermore, other publications using ABCD data have set a precedent of including these covariates in their analyses [19, 24, 35].

We first generated separate models that each contained a single measure of SES (ADI, PE, or HI) along with covariates to individually assess the relationship between each of these measures and our outcome variables. These models were used for further analyses investigating the influence of BMI on the relationships. PE and HI were dummy coded categorical variables with Less than High School and <50 K acting as the respective reference categories. All three measures were then implemented into expanded models to maximize the amount of variance potentially explained by SES. Calculation of the generalized variation inflation factors for each of our predictors did not demonstrate the presence of substantial multicollinearity in our extended models (max gVIF = 1.951), indicating that the inclusion of all measures of SES in these models was not problematic for the interpretation of regression coefficients [55]. We also included physical activity as an additional covariate in these models to account for any variance potentially explained by levels of fitness. This was done after investigating the influence of fitness levels on BMI using an additional model with physical activity as the independent variable of interest. All of the data meet the assumptions of these models. While the distribution of the error terms of the SES vs BMI model was not fully normal, the assumption of normality can be relaxed for large sample sizes due to the central limit theorem [56].

It is important to account for site differences in the ABCD sample, especially with regards to imaging measures. However, because ADI is based on geographic location, ADI and study site are highly confounded in the ABCD sample (*F* = 158.4, *P* < 0.001). As this confound makes it difficult to disentangle the individual effects of ADI and study site on our outcome measures, we did not include the study site in our primary regression models. However, we did generate supplementary linear mixed effect models that include site location as a random effect using the lmer function from the lme4 package in R [57]. The initial calculation of the intraclass correlation coefficients for our outcome measures indicated that the proportion of the variance attributed to the study site was minimal (Table S1). However, as a low ICC does not necessarily mean a multilevel approach is unwarranted, we still calculated the mixed effect models. Including the study site as a random effect abolishes the significant relationships of our morphological measures with ADI, but not with HI or PE (which are not inherently tied to location).

Race/ethnicity is also strongly related to ABCD site location [chi-squared test for independence of categorical variables; χ^2^(60) = 2026.1, *P* < 0.001; *N* = 7607], and the confound between race/ethnicity and socioeconomic status is well-documented in the US [58]. Thus, we also generated additional multilevel models with site as a random effect that did not include race/ethnicity as covariates. Notably, our morphological measures were once more significantly related to ADI in these models. Results for all SES variables in our additional mixed effect models can be found in the supplementary materials (Tables S2-S4).

### Comparing correlated coefficients

We also sought to determine if SES associations were specific to certain domains of cognitive function. To assess the differential association of the three NIH Toolbox subtests with ADI, we compared the correlation coefficients (Pearson’s R) of each direct relationship using the paired.r function from the “psych” package in R [59]. This command generates a t-test of the difference between two dependent correlations by first converting the correlation coefficients to z-scores using a Fisher’s R to Z transformation [60].

### Assessing the role of BMI in the relationship between SES and neurocognition/cortical morphology

Traditional mediation analysis requires either a continuous or binary categorical explanatory variable [50]. As ADI is continuous, we investigated the mediating role of BMI in the relationships between ADI and our independent variables of interest using the “mediation” package in R [61]. Because ADI was the explanatory variable of interest, we used models with ADI as the sole measure of SES to keep our analyses as parsimonious as possible. In the context of mediation models, the direct and total effects refer to the beta coefficients of the explanatory variable of interest in models with and without the mediator respectively. In these models, the indirect effect is equal to the reduction in the direct effect of the primary variable of interest on the dependent variable due to a third, mediating variable and is the quantification of the amount of mediation. 1000 Monte Carlo Simulations were used to generate Quasi-Bayesian 95% CI for the average indirect (mediating) effects, the average direct effects, and the total effects [62].

As PE and HI are non-binary categorical variables, mediation analyses were not conducted with these variables. Rather, we investigated the possible moderating role of BMI in the relationship between these variables and composite neurocognition and total cortical volume by testing for interaction effects between BMI and the different categorical levels of PE and HI in the respective individual models.

## Results

The current study used a sample of 7607 subjects (age = 9.91 ± 0.613 years, 52.9% male). Descriptive demographic information of the study sample can be found in Table 1. Overall, greater SES was associated with lower BMI, higher composite neurocognitive scores, and greater total cortical volume. BMI played a significant mediating role in the relationship between ADI and both neurocognitive scores and total cortical volume.

**Table 1:** Participant characteristics.

Demographic	No. of participants^a^ (%) (*N* = 7607)
*Sex*	
Male	4023 (52.9)
Female	3584 (47.1)
*Race*	
White	5285 (69.5)
Black	1408 (18.5)
Asian	478 (6.28)
Other	436 (5.72)
*Ethnicity*	
Non-Hispanic	6053 (79.6)
Hispanic	1554 (20.4)
ADI (Mean, SD)	92.15 (24.6) Range: 0–125.75
*Annual household income*	
<50 K	2227 (29.3)
50 K - <100 K	2186 (28.7)
≤100 K	3194 (42.0)
*Highest level of parental education*	
Less than High School	411 (5.40)
High School Graduate/GED	586 (7.70)
Some College	918 (12.1)
Associates/Bachelor’s Degree	2947 (38.7)
Postgraduate Degree	2745 (36.1)
*BMI*^**b**^	
Mean (SD)	18.6 (3.85)
Underweight (<5%)	301 (3.96)
Healthy Range (5% - <85%)	5044 (66.3)
Overweight (85% - <95%)	1129 (14.8)
Obese (≥95%)	1133 (14.9)

**a** All subjects are adolescents with a mean sample age of 9.91 ± 0.621 years.

**b** Weight Status categories based on CDC guidelines. National BMI percentiles were determined using CDC BMI-for-Age Growth Charts for boys and for girls. Age of 10 years was used (Centers for Disease Control and Prevention).

### SES is associated with BMI, composite neurocognitive score, and total cortical volume in multiple linear regression models

In our individual models, greater ADI was significantly related to greater BMI (**β** = 0.0135, *P* < 0.001), lower total cortical volume (**β** = −57.3, *P* < 0.001), and lower composite neurocognitive scores (**β** = −0.105, *P* < 0.001), establishing the relationships necessary for mediation analysis. Each category of increasing SES for HI was significantly related to lower BMI, greater composite neurocognitive scores, and greater total cortical volume. Additionally, every category of PE except “High School Grad/GED” was associated with lower BMI and greater neurocognitive scores. However, only “Postgraduate Degree” was significantly related to increased total cortical volume (Tables S5–S7).

**Fig. 1:**
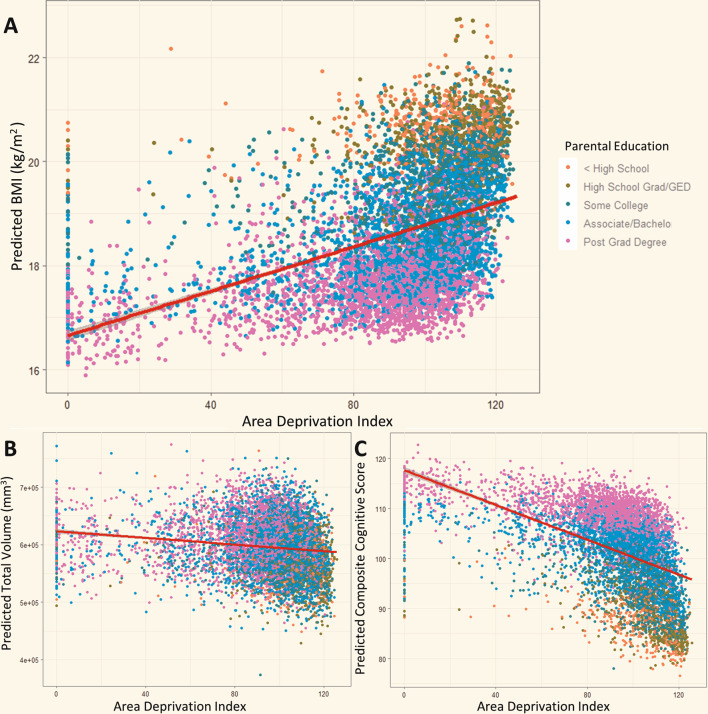
ADI is associated with predicted BMI, cortical volume, and neurocognitive score. ADI vs predicted values of (**A**) BMI, (**B**) Cortical Volume, and (**C**) Composite Neurocognitive Score. Extended linear regression models follow the formula: **Dependent Variable** = **β**_ADI_ **ADI** + **β**_PE_ **PE** + **β**_HI_ **HI** + **β**_PA_ **Physical Activity** + **β**_Age_ **Age** + **β**_Race_ **Race** + **β**_Ethnicity_ **Ethnicity** + **β**_sex_ **Sex** + **β**_IV_ **Intracranial Volume** + **β**_0_. The *x*-axis position of each data point is determined by the corresponding subject’s ADI value. The position on the *y*-axis corresponds to the resultant dependent variable value after the independent variable values of a given subject are inserted into the equation above. The curve on each graph represents the line of best fit for predicted values vs ADI using ordinary least squares regression and demonstrates the direction of the relationships between ADI and BMI, Cortical Volume, and Neurocognitive Score (positive, negative, and negative respectively). The color of each data point represents the corresponding subject’s highest level of parental education.

Largely, the significance and direction of these relationships remained consistent in our extended models that incorporated all of our measures of SES simultaneously for BMI and composite neurocognitive score. However, only “Postgraduate Degree” and annual household income greater than 100 K were significantly associated with total cortical volume in the extended model. (Tables S5–S7). The directions of these relationships are visualized in Fig. 1 using scatter plots of ADI vs predicted response variable measures based on the extended regression models. Additionally, the model predicting BMI based on physical activity in the week prior to the interview indicated that physical activity was negatively correlated with BMI (**β** = −0.084, *P* < 0.001) (Table S8).

In the models that included interaction terms between BMI and the different levels of PE and HI individually, the only significant interaction was between BMI and annual household income of 50-<100 K (**β** = −0.27, *P* = 0.044) for neurocognitive score (Table S9, S10). Full statistical summary data of all models can be found in the Supplement.

### SES is associated with specific cortical regions and neurocognitive subdomains in multiple linear regression models

Because greater BMI has previously been associated with impaired executive function specifically, we investigated the relationship of SES with prefrontal cortex volume as well as with three NIH Toolbox subtests assessing different components of executive function. We also included an analysis of the relationship between SES and occipital cortex volume as a reference region of interest to compare with our prefrontal cortical analysis.

In our individual models, ADI was significantly associated with lower volume in our primary region of interest, prefrontal cortex (**β** = −15.3, *P* = 0.003), but not our control region, occipital cortex (**β** = −2.92, *P* = 0.206). In the individual models of PE and HI, only “Postgraduate Degree” was significantly related to greater prefrontal cortex volume, while none of the measures of parental education were significantly related to occipital cortex volume. Both levels of increasing HI were associated with greater volume in both regions (Table S11, S12). In the extended model predicting prefrontal volume, the relationships with ADI and “50-<100 K” were no longer significant. Full statistical summary data of all models can be found in the Supplement.

**Fig. 2:**
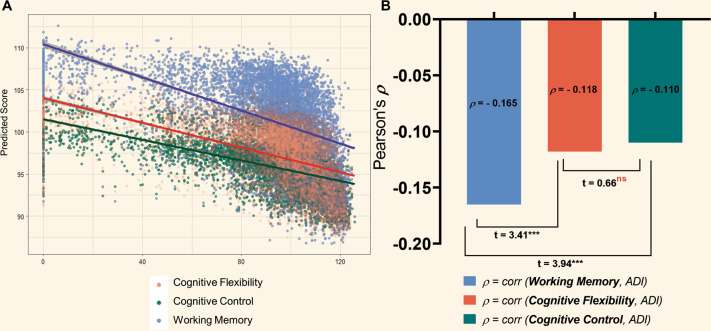
Neurocognitive subtests of different domains of executive function are differentially associated with ADI. **A** ADI vs. predicted values on the Flanker (Cognitive Control), Card Sort (Cognitive Flexibility), and List Sort (Working Memory) tasks based on our extended linear regression models as described in Fig. 1. **B** Statistic comparison of correlation coefficients. Bars indicate magnitude of Pearson’s correlation coefficient (**ρ**) for ADI and each subtest. Fisher’s R-to-Z transformation was used to generate a t-test statistic for the difference between each pair of correlations. The correlation between ADI and Working Memory (List Sort Task) was significantly greater in magnitude than the correlations with the other two tasks. Cognitive Flexibility (Card Sort) and Cognitive Control (Flanker Task) correlations with ADI were not significantly different from one another.

Greater ADI was significantly associated with lower scores on all three neurocognitive subtests (Flanker [Cognitive Control]: **β** = −0.0411, *P* < 0.001; List Sort [Working Memory]: **β** = −0.0616, *P* < 0.001; Card Sort [Cognitive Flexibility]: **β** = −0.0439, P < 0.001). The direction of these relationships are visualized in Fig. 2A. Each category of increasing SES for PE and HI was significantly associated with better performance on all three subtests except “High School/GED” on all three tasks, and “Some College” on the Card Sort task. All relationships remained true in the extended models as shown in the Supplement (Tables S13–S15).

Analyses of regression models where ADI predicted neurocognitive subtests scores indicated that all three models significantly fit the data (Flanker: *F*_8,7598_ = 27.49, *P* < 0.001; List Sort: *F*_8,7598_ = 84.02, *P* < 0.001; Card Sort:: *F*_8,7598_ = 41.27, *P* < 0.001). However, as the F-test statistic and Beta-coefficient for ADI were greater in magnitude for the List Sort task, we used a Fisher’s R-to-Z transformation to statistically compare the correlations of ADI with the different subtests. Pearson’s correlation coefficients of ADI with the Flanker, List Sort, and Card Sort Subtests were **ρ** = −0.110, **ρ** = −0.165, **ρ** = −0.118, respectively. Comparison of correlated coefficients revealed that the correlation between ADI and scores on the List Sort task was significantly greater in magnitude than the correlation of ADI with the Flanker (*t* = 3.94, *P* < 0.001) and Card Sort (*t* = 3.41, *P* < 0.001) tasks. Additionally, the correlations with the Flanker task and the Card Sort task were not significantly different from each other (*t* = 0.66, *P* = 1.00) (Fig. 2B).

### Mediation analysis of ADI, BMI and neurocognition/cortical volume

**Fig. 3:**
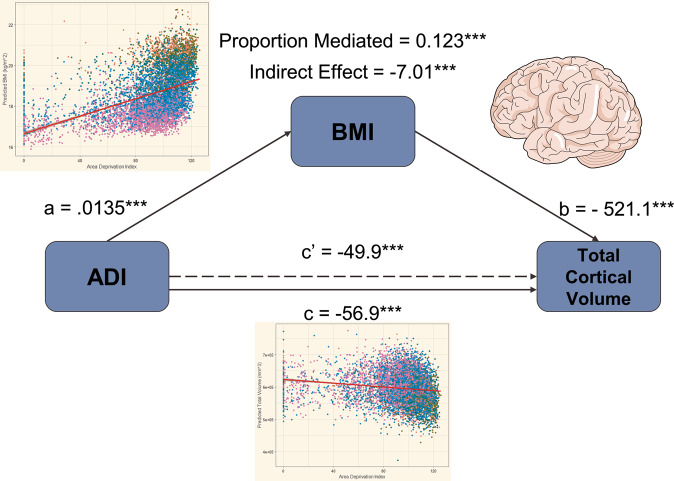
Mediation model demonstrating the significant indirect effect of BMI in the relationship between ADI and total cortical volume. Reported values of *a*, *b*, and *c* are regression coefficients for each path. The indirect effect is the product of paths a and b and is described as the amount of mediation attributed to BMI. The dashed line corresponding to *c*′ indicates the reduced direct effect of ADI on Cortical Volume when BMI is included as a mediator. Inset graphs are from Fig. 1 and are used to illustrate the relationships that must be established to conduct mediation analysis.

**Fig. 4:**
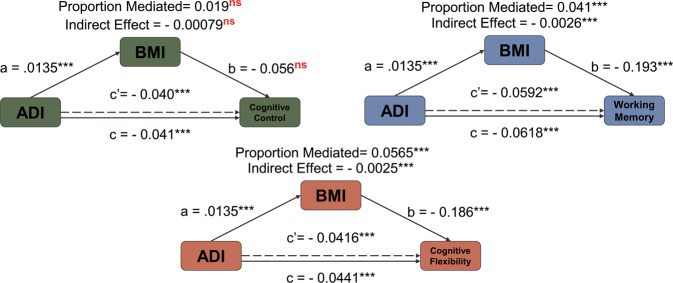
Mediation models of demonstrating indirect effect of BMI in the relationship between ADI and neurocognitive subtests. Model Characteristics are identical to those described in Fig. 3, without insets. The indirect effects of BMI are significant for the Card Sort and List Sort tasks, but not for the Flanker task.

In the following models, ADI is the primary independent variable of interest, with BMI serving as the potential mediator responsible for the indirect effect. Significant indirect effects attributed to BMI were associated with the relationship between ADI and both total cortical volume (Indirect Effect = −7.01, *P* < 0.001) (Fig. 3) and prefrontal cortex volume (Indirect Effect = −2.58, *P* < 0.001). Mediation analysis of the relationship between ADI and occipital cortex volume is unnecessary because our multiple linear regression model did not indicate a significant association between these two variables. BMI also partially mediated the relationship between ADI and Composite Neurocognitive Score, with a significant indirect effect of −0.0039 (*P* < 0.001). The indirect effect of BMI was significant in the relationship between ADI and both the Card Sort (Indirect Effect = −0.0025, *P* < 0.001) and List Sort (Indirect Effect = −0.0026, *P* < 0.001) tasks, but not the Flanker Task (Indirect Effect = −0.00079, *P* = 0.17) (Fig. 4). Full statistical summary data can be found in the Supplement (Table S16).

## Discussion

The results of the present study broadly indicated a significant negative association between measures of SES (PE, ADI, HI) and BMI, as well as significant positive associations between SES measures and both neurocognition and cortical volume in a diverse sample of 7607 9–10-year-old children. These results are consistent with prior findings [29, 63]. The relationship between SES and cortical brain volume differed across brain regions (prominent in prefrontal but not occipital cortex). Additionally, SES appears to have a differential role across domains of executive function (with a more prominent impact on working memory than cognitive control and cognitive flexibility). Notably, BMI had a significant indirect effect in the associations between ADI and various measures of brain structure and cognition.

BMI was significantly associated with all SES measures we assessed, barring the “High School Grad/GED” category of PE. At the neighborhood level, a greater ADI value predicted greater BMI, while categories of increasing SES for both HI and PE predicted lower BMI at the individual level. Several studies had previously shown associations of childhood obesity with both household income and parental education, citing the affordability of highly processed, calorically dense foods, as well as different cultural attitudes among educated families towards healthy dietary habits and obesity as possible causal factors [9, 52, 64, 65]. Childhood obesity is also associated with neighborhood SES [66]. This relationship has partially been attributed to the higher density of unhealthy fast-food outlets in low-income areas and low access to healthy foods (food deserts) [67–69]. Additionally, fewer recreational facilities and higher crime rates in deprived neighborhoods contribute to decreased physical activity and subsequent increased obesity among children residing in these areas [70].

Greater total and prefrontal cortical volumes were observed with greater SES, though the exact nature of this relationship is unclear. Prior studies using this dataset have shown that higher SES at the neighborhood level is positively correlated with greater cortical thickness in regions of the prefrontal cortex as well as greater hippocampal volume, consistent with our findings [29, 33]. Several studies have shown that greater cortical volume and gray matter thickness in certain brain regions correlate with greater parental education, especially in the prefrontal cortex [25, 71, 72]. However, in the present study only the “Postgraduate Degree” educational category was significantly associated with total and prefrontal cortical volumes. Sample sizes in the cited publications were limited in comparison to that of the ABCD dataset (largest sample of 283) and the large sample size of the present study may allow for a more accurate representation of the relationship between parental education and cortical structure.

SES was positively associated with composite neurocognitive performance, as well as performance on subtests assessing specific domains of executive function. These results are consistent with previous studies describing the relationship between SES and neurocognition/executive function on both the individual and neighborhood level [32, 73–75]. Further investigation revealed that the negative correlation between ADI and the List Sort Task measuring working memory was significantly greater in magnitude than that of the other two subtests. Prior studies similarly observed stronger associations between SES and working memory than SES and cognitive control, though these studies did not assess cognitive flexibility [30, 76]. However, Sarsour et al. [77] did investigate the association between SES and the three cognitive domains assessed in the present study in 60 children, and found that the impact of SES was weakest on working memory. Differences in sample size and demographics as well as differences in tasks used to measure cognitive domains may account for these discrepancies. Chronic stress in childhood as well as enrichment of the childhood home environment have been shown to partially mediate the relationship between SES and working memory [78–80]. This finding is particularly notable since working memory is considered a foundational cognitive function, providing the basis for many higher-order cognitive processes [81]. However, the reason working memory would be affected more than cognitive flexibility or cognitive control is unclear. It is likely that these domains of executive function have distinct neural correlates within prefrontal circuits. For example, the dorsolateral prefrontal cortex is critical for working memory, while it is the anterior prefrontal and anterior cingulate regions that are activated during the Flanker task [80, 82, 83]. Therefore, future studies specifically examining how each of these regions relate to SES in the context of various cognitive domains could provide more insight.

Our results indicated that BMI partially mediated the relationships between neighborhood SES as measured by ADI and both neurocognition and cortical volume. Several neurobiological consequences of high BMI could potentially lead to lower cortical volume. The brain relies on the cerebrovascular system to supply oxygen and glucose necessary for healthy neuronal function and vascular dysfunction in the brain can lead to reduced cerebral perfusion and subsequent neuronal atrophy [84]. Childhood obesity has been associated with endothelial dysfunction, and it was suggested that deficits in endothelial-dependent vasodilation may lead to inadequate glucose supply in the brain and a suboptimal neuronal environment [85, 86]. Furthermore, it is also possible that some ABCD participants who were obese might have had some level of insulin resistance, hypoglycemia, increases in inflammatory signals and/or cardiac disruption, all of which could negatively affect brain development [87–90]. Further, obese children are at a higher risk of sleep apnea, which in turn could negatively affect brain development and cognition [91, 92]. Lastly, evidence in animal models has shown that weight gain can lead to synaptic loss as a result of decreased dendritic spine density and synaptic protein expression [93].

Many of these neuronal consequences of elevated BMI could also partially explain its role as a mediator between SES and neurocognition. For instance, increases in brain activation are accompanied by increased blood flow and glucose and oxygen consumption in the activated regions [94, 95]; therefore obesity-related endothelial dysfunction and sub-optimal blood flow may also contribute to cognitive impairments [86]. Deficits in cortical activation itself could also underlie impaired neurocognition related to BMI. fMRI studies have shown obesity-related differences in cortical activation during food-related cognitive tasks and in response to food images [96, 97]. Furthermore, several studies showing deficits in executive function related to BMI have cited cortical structural alterations similar to the ones observed in the present study [14, 98]. If higher BMI is driving brain structural deficits, which may or may not manifest in cognitive dysfunction later on, this might explain why in the present study we observed a greater proportion of SES-cortical structure relationships mediated by BMI, in comparison to the number of SES-cognition models.

On a much broader scale, a variety of sociological factors might explain part of the relationship between childhood obesity and cognitive performance. Regular exercise and participation in school sports have been positively associated with academic achievement and executive function [99, 100]. Further, exercise as an interventional measure can improve executive function performance and academic outcomes in overweight children [101]. Evidence suggests that rates of participation in school sports are lower among obese children, though the direction of this relationship is unclear [102, 103]. As our results indicated that physical activity is negatively correlated with BMI, future investigations parsing the contributions of fitness and BMI to neurodevelopmental outcomes may be useful. The larger likelihood of bullying victimization among obese children could also account for part of the association between elevated BMI and cognition, as victims of bullying tend to have poorer academic and executive function performance [104–107].

Several limitations exist in our study, and therefore, our results must be interpreted with care. Because of the cross-sectional nature of the study, it is not possible to draw conclusions on the direction of associations. While the present models consider BMI to be the mediating variable, reverse causality is also possible, where cognition or cortical volume facilitate the relationship between SES and BMI. Additionally, SES could drive changes in BMI and developmental measures independently, suggesting a joint causative model. As such, we do not claim that our data shows explicit evidence of causation, but that our findings lend support to a hypothesis that may be further explored in longitudinal studies. Future studies may also consider more complex mediation models beyond the traditional methods that explore exposure-mediator interactions or categorical independent variables. Additionally, since the HI data were collected via questionnaires that had differing increments of income between response options, income could not effectively be modeled as a continuous variable. Income is inherently a continuous variable and future studies should collect actual income levels to model it as such. Furthermore, Fisher’s R-to-Z transformation allows for the statistical comparison of correlation coefficients of only direct relationships that do not account for covariates. Lastly, the relationships between our morphological measures and ADI are no longer significant when including study site as a random effect and race/ethnicity as covariates in mixed-effect models. However, ADI is calculated based on location and there is an inherent overlap in the meaningful variance in our outcome measures explained by study site and ADI. Furthermore, geographic location, race/ethnicity, and SES are highly confounded making it difficult to parse the individual effects of these variables on outcome measure. Future longitudinal studies are necessary to disentangle the complex associations between these variables.

In conclusion, SES was positively associated with both cortical volume and neurocognitive performance. These relationships were distinct across brain regions and domains of executive function, with working memory having a significantly stronger correlation with ADI than the other two domains. Evidence from this study suggests that the relationships between ADI and both cortical volume and neurocognition could be partially mediated by BMI. While we do not infer causation, these findings suggest a hypothesis on which future longitudinal studies could be based. Such studies could further elucidate the exact directionality of these associations and support interventional measures aimed at facilitating healthy body weight to improve outcomes in brain development in low SES populations.

## Supplementary information

Supplement

## Data Availability

The R code used to conduct this analysis will be made available upon reasonable request. Data used in the preparation of this article were obtained from the Adolescent Brain Cognitive DevelopmentSM (ABCD) Study (https://abcdstudy.org), held in the NIMH Data Archive (NDA). This is a multisite, longitudinal study designed to recruit more than 10,000 children age 9–10 and follow them over 10 years into early adulthood. The ABCD Study^®^ is supported by the National Institutes of Health and additional federal partners under award numbers U01DA041048, U01DA050989, U01DA051016, U01DA041022, U01DA051018, U01DA051037, U01DA050987, U01DA041174, U01DA041106, U01DA041117, U01DA041028, U01DA041134, U01DA050988, U01DA051039, U01DA041156, U01DA041025, U01DA041120, U01DA051038, U01DA041148, U01DA041093, U01DA041089, U24DA041123, U24DA041147. A full list of supporters is available at https://abcdstudy.org/federal-partners.html. A listing of participating sites and a complete listing of the study investigators can be found at https://abcdstudy.org/consortium_members/. ABCD consortium investigators designed and implemented the study and/or provided data but did not necessarily participate in analysis or writing of this report. This manuscript reflects the views of the authors and may not reflect the opinions or views of the NIH or ABCD consortium investigators. The ABCD data repository grows and changes over time. The ABCD data used in this report came from 10.15154/1503209.

## Code availability

The R code used to conduct this analysis will be made available upon reasonable request.
