# Supplementary material for: Socioeconomic status, BMI, and brain development in children
Source: Transl Psychiatry. 2022 Jan 24;12:33. doi: 10.1038/s41398-022-01779-3 (PMC8786961; doi:10.1038/s41398-022-01779-3)
Supplement: Supplementary file 1 — Supplement [file 41398_2022_1779_MOESM1_ESM.docx]

| **Table S1: Intraclass Correlation Coefficient for Study Site as a Random Effect** | |
| --- | --- |
| *Measures* | *ICC* |
| **BMI** | 0.0378 |
| **Total Volume** | 0.0349 |
| **Prefrontal Volume** | 0.0397 |
| **Occipital Volume** | 0.0467 |
| **Composite Neurocognition** | 0.0536 |
| **Flanker Task** | 0.0178 |
| **List Sort Task** | 0.0294 |
| **Card Sort Task** | 0.0192 |

| **Table S2: Relationships between ADI and Outcome Measures in Linear Mixed Effect Models** | | | | |
| --- | --- | --- | --- | --- |
| *Measures* | **ADI (Mixed Effect Model with Site as Random Effect)** | | **ADI (Mixed Effect Model with Site as Random Effect, without Race/Ethnicity)** | |
|  | *Estimate* | *p* | *Estimate* | *p* |
| **BMI** | 0.0155 | **<0.001** | .0252 | **<0.001** |
| **Total Volume** | -27.31 | 0.0912 | -100.9 | **<0.001** |
| **Prefrontal Volume** | -4.744 | 0.3549 | -18.94 | **<0.001** |
| **Occipital Volume** | -3.004 | 0.2730 | -14.462 | **<0.001** |
| **Composite Neurocognition** | -0.0986 | **<0.001** | -0.1539 | **<0.001** |
| **Flanker Task** | -0.0360 | **<0.001** | -0.0495 | **<0.001** |
| **List Sort Task** | -.0631 | **<0.001** | -0.0919 | **<0.001** |
| **Card Sort Task** | -0.0382 | **<0.001** | -0.0603 | **<0.001** |

| **Table S3: Relationships between HI and Outcome Measures in Linear Mixed Effect Models** | | | | |
| --- | --- | --- | --- | --- |
| *Measures* | **HI (Mixed Effect Model with Site as Random Effect)** | | **HI (Mixed Effect Model with Site as Random Effect, without Race/Ethnicity)** | |
|  | *Estimate* | *p* | *Estimate* | *p* |
| **BMI** |  |  |  |  |
| *50-<100K* | -0.6426 | **<0.001** | -1.053 | **<0.001** |
| *>=100K* | -1.273 | **<0.001** | -1.929 | **<0.001** |
| **Total Volume** |  |  |  |  |
| *50-<100K* | 3,219 | **<0.001** | 6,941 | **<0.001** |
| *>=100K* | 5,251 | **<0.001** | 10,765 | **<0.001** |
| **Prefrontal Volume** |  |  |  |  |
| *50-<100K* | 855.6 | **0.0032** | 1,449 | **<0.001** |
| *>=100K* | 1,357 | **<0.001** | 2,315 | **<0.001** |
| **Occipital Volume** |  |  |  |  |
| *50-<100K* | 208.1 | 0.1455 | 878.6 | **<0.001** |
| *>=100K* | 380.3 | **.0090** | 1334 | **<0.001** |
| **Composite Neurocognition** |  |  |  |  |
| *50-<100K* | 6.632 | **<0.001** | 8.937 | **<0.001** |
| *>=100K* | 9.838 | **<0.001** | 13.271 | **<0.001** |
| **Flanker Task** |  |  |  |  |
| *50-<100K* | 2.341 | **<0.001** | 2.855 | **<0.001** |
| *>=100K* | 3.189 | **<0.001** | 3.891 | **<0.001** |
| **List Sort Task** |  |  |  |  |
| *50-<100K* | 3.885 | **<0.001** | 5.076 | **<0.001** |
| *>=100K* | 6.117 | **<0.001** | 7.871 | **<0.001** |
| **Card Sort Task** |  |  |  |  |
| *50-<100K* | 2.888 | **<0.001** | 3.873 | **<0.001** |
| *>=100K* | 3.939 | **<0.001** | 5.397 | **<0.001** |

| **Table S4: Relationships between PE and Outcome Measures in Linear Mixed Effect Models** | | | | |
| --- | --- | --- | --- | --- |
| *Measures* | **PE (Mixed Effect Model with Site as Random Effect)** | | **PE (Mixed Effect Model with Site as Random Effect, without Race/Ethnicity)** | |
|  | *Estimate* | *p* | *Estimate* | *p* |
| **BMI** |  |  |  |  |
| *High School Grad* | -0.1208 | 0.6931 | -0.2177 | 0.4174 |
| *Some College* | -0.5627 | **0.0224** | -0.8587 | **<0.001** |
| *Associate's/Bachelor's* | -1.284 | **<0.001** | -1.857 | **<0.001** |
| *Postgraduate Degree* | -1.853 | **<0.001** | -2.612 | **<0.001** |
| **Total Volume** |  |  |  |  |
| *High School Grad* | -224.1 | 0.8953 | -1,223 | 0.4801 |
| *Some College* | 2,817 | 0.1067 | 3,451 | **0.0420** |
| *Associate's/Bachelor's* | 3,567 | **0.0245** | 6,717 | **<0.001** |
| *Postgraduate Degree* | 6,387 | **<0.001** | 11,032 | **<0.001** |
| **Prefrontal Volume** |  |  |  |  |
| *High School Grad* | 264.4 | 0.6931 | 490.3 | 0.4309 |
| *Some College* | 1,122 | 0.0569 | 1,661 | **0.0034** |
| *Associate's/Bachelor's* | 1,104 | **0.0433** | 2,053 | **<0.001** |
| *Postgraduate Degree* | 1,991 | **<0.001** | 3,186 | **<0.001** |
| **Occipital Volume** |  |  |  |  |
| *High School Grad* | -460.3 | 0.1514 | -809.9 | **0.0098** |
| *Some College* | -221.3 | 0.4824 | -302.5 | 0.322 |
| *Associate's/Bachelor's* | 79.47 | 0.7742 | 439.4 | 0.0947 |
| *Postgraduate Degree* | 117.4 | 0.6931 | 734.5 | **0.0057** |
| **Composite Neurocognition** |  |  |  |  |
| *High School Grad* | 2.726 | **0.0166** | 2.454 | **0.0263** |
| *Some College* | 6.096 | **<0.001** | 6.882 | **<0.001** |
| *Associate's/Bachelor's* | 10.47 | **<0.001** | 12.74 | **<0.001** |
| *Postgraduate Degree* | 15.75 | **<0.001** | 18.96 | **<0.001** |
| **Flanker Task** |  |  |  |  |
| *High School Grad* | 0.8639 | 0.4022 | 0.6889 | 0.4454 |
| *Some College* | 2.974 | **<0.001** | 3.066 | **<0.001** |
| *Associate's/Bachelor's* | 3.640 | **<0.001** | 4.087 | **<0.001** |
| *Postgraduate Degree* | 4.577 | **<0.001** | 5.274 | **<0.001** |
| **List Sort Task** |  |  |  |  |
| *High School Grad* | 2.235 | **0.0245** | 1.866 | 0.0511 |
| *Some College* | 4.629 | **<0.001** | 4.772 | **<0.001** |
| *Associate's/Bachelor's* | 6.759 | **<0.001** | 7.682 | **<0.001** |
| *Postgraduate Degree* | 10.11 | **<0.001** | 11.51 | **<0.001** |
| **Card Sort Task** |  |  |  |  |
| *High School Grad* | 0.9576 | 0.4022 | 0.8076 | 0.4309 |
| *Some College* | 1.923 | 0.0522 | 2.231 | **0.0186** |
| *Associate's/Bachelor's* | 3.547 | **<0.001** | 4.515 | **<0.001** |
| *Postgraduate Degree* | 5.711 | **<0.001** | 7.103 | **<0.001** |

| **Table S5: Model Output for SES Predicting BMI** | | | | | | | | |
| --- | --- | --- | --- | --- | --- | --- | --- | --- |
|  | **BMI (Extended)** | | **BMI (ADI)** | | **BMI (PE)** | | **BMI (HI)** | |
| *Predictors* | *Estimate (95% CI)* | *p* | *Estimate (95% CI)* | *p* | *Estimate (95% CI)* | *p* | *Estimate (95% CI)* | *p* |
| Intercepts | 11.336 (9.636 – 13.037) | **<0.001** | 9.842 (8.161 – 11.522) | **<0.001** | 12.289 (10.638 – 13.940) | **<0.001** | 11.506 (9.882 – 13.129) | **<0.001** |
| ADI | 0.007 (0.003 – 0.010) | **<0.001** | 0.013 (0.010 – 0.017) | **<0.001** |  |  |  |  |
| High School Grad/GED | -0.079 (-0.548 – 0.391) | 0.743 |  |  | -0.100 (-0.570 – 0.371) | 0.678 |  |  |
| Some College | -0.502 (-0.943 – -0.061) | **0.052** |  |  | -0.619 (-1.058 – -0.180) | **0.011** |  |  |
| Associate's/Bachelor's Degree | -1.061 (-1.478 – -0.643) | **<0.001** |  |  | -1.383 (-1.784 – -0.982) | **<0.001** |  |  |
| Postgraduate Degree | -1.357 (-1.802 – -0.913) | **<0.001** |  |  | -1.905 (-2.317 – -1.492) | **<0.001** |  |  |
| 50-<100K | -0.317 (-0.563 – -0.071) | **0.019** |  |  |  |  | -0.744 (-0.974 – -0.514) | **<0.001** |
| >=100K | -0.663 (-0.933 – -0.394) | **<0.001** |  |  |  |  | -1.361 (-1.588 – -1.133) | **<0.001** |
| Physical Activity | -0.054 (-0.091 – -0.018) | **0.009** |  |  |  |  |  |  |
| Race (Black) | 1.293 (1.052 – 1.534) | **<0.001** | 1.800 (1.572 – 2.028) | **<0.001** | 1.531 (1.300 – 1.763) | **<0.001** | 1.464 (1.225 – 1.703) | **<0.001** |
| Race (Asian) | -0.111 (-0.460 – 0.237) | 0.532 | -0.140 (-0.492 – 0.212) | 0.437 | -0.217 (-0.563 – 0.128) | 0.218 | -0.246 (-0.592 – 0.101) | 0.164 |
| Race (Other) | 0.374 (-0.016 – 0.764) | 0.242 | 0.813 (0.426 – 1.201) | **<0.001** | 0.424 (0.033 – 0.815) | **0.134** | 0.566 (0.178 – 0.955) | **0.017** |
| Ethnicity (Hispanic) | 1.161 (0.929 – 1.393) | **<0.001** | 1.604 (1.380 – 1.828) | **<0.001** | 1.267 (1.037 – 1.498) | **<0.001** | 1.311 (1.082 – 1.541) | **<0.001** |
| Sex (Female) | 0.271 (0.088 – 0.454) | **0.005** | 0.214 (0.029 – 0.398) | **0.031** | 0.267 (0.084 – 0.451) | **0.006** | 0.245 (0.061 – 0.429) | **0.012** |
| Age | 0.046 (0.035 – 0.057) | **<0.001** | 0.046 (0.035 – 0.057) | **<0.001** | 0.043 (0.032 – 0.055) | **<0.001** | 0.046 (0.035 – 0.057) | **<0.001** |
| Intracranial Volume | 0.000 (0.000 – 0.000) | **<0.001** | 0.000 (0.000 – 0.000) | **0.009** | 0.000 (0.000 – 0.000) | **<0.001** | 0.000 (0.000 – 0.000) | **<0.001** |
| R^2^ / R^2^ adjusted | 0.102 / 0.100 | | 0.081 / 0.080 | | 0.095 / 0.094 | | 0.090 / 0.089 | |

| **Table S6: Model Output for SES Predicting Total Cortical Volume** | | | | | | | | |
| --- | --- | --- | --- | --- | --- | --- | --- | --- |
|  | **Cortical Volume (Extended)** | | **Cortical Volume (ADI)** | | **Cortical Volume (PE)** | | **Cortical Volume (HI)** | |
| *Predictors* | *Estimate (95% CI)* | *p* | *Estimate (95% CI)* | *p* | *Estimate (95% CI)* | *p* | *Estimate (95% CI)* | *p* |
| Intercepts | 261806.460 (247353.522 – 276259.397) | **<0.001** | 266244.157 (252072.520 – 280415.793) | **<0.001** | 258311.651 (244318.842 – 272304.460) | **<0.001** | 259112.673 (245373.351 – 272851.995) | **<0.001** |
| ADI | -23.800 (-55.147 – 7.547) | 0.182 | -57.251 (-87.085 – -27.417) | **<0.001** |  |  |  |  |
| High School Grad/GED | -3349.629 (-7336.614 – 637.357) | 0.199 |  |  | -3203.632 (-7190.316 – 783.052) | 0.230 |  |  |
| Some College | 410.284 (-3339.699 – 4160.267) | 0.830 |  |  | 1061.713 (-2656.650 – 4780.076) | 0.594 |  |  |
| Associate's/Bachelor's Degree | 617.099 (-2930.239 – 4164.436) | 0.757 |  |  | 2278.857 (-1120.515 – 5678.230) | 0.216 |  |  |
| Postgraduate Degree | 3982.853 (205.602 – 7760.105) | **0.044** |  |  | 6647.753 (3153.994 – 10141.511) | **<0.001** |  |  |
| 50-<100K | 2014.348 (-79.359 – 4108.054) | 0.079 |  |  |  |  | 3535.475 (1589.691 – 5481.260) | **<0.001** |
| >=100K | 3435.793 (1145.632 – 5725.955) | **0.013** |  |  |  |  | 6412.202 (4484.012 – 8340.393) | **<0.001** |
| Physical Activity | 217.483 (-91.009 – 525.976) | 0.267 |  |  |  |  |  |  |
| Race (Black) | -23207.408 (-25254.387 – -21160.429) | **<0.001** | -25623.068 (-27545.820 – -23700.317) | **<0.001** | -24330.299 (-26291.609 – -22368.989) | **<0.001** | -23948.556 (-25969.871 – -21927.241) | **<0.001** |
| Race (Asian) | -3564.890 (-6525.935 – -603.844) | **0.024** | -3365.239 (-6333.580 – -396.898) | **0.035** | -3173.116 (-6101.452 – -244.780) | **0.045** | -2964.246 (-5893.507 – -34.986) | **0.054** |
| Race (Other) | 1430.416 (-1886.626 – 4747.457) | 0.636 | -266.243 (-3532.988 – 3000.502) | 0.873 | 1151.313 (-2162.152 – 4464.779) | 0.593 | 919.352 (-2369.475 – 4208.179) | 0.667 |
| Ethnicity (Hispanic) | -4655.936 (-6629.967 – -2681.905) | **<0.001** | -6473.113 (-8361.290 – -4584.936) | **<0.001** | -5165.683 (-7117.892 – -3213.473) | **<0.001** | -5072.507 (-7014.695 – -3130.319) | **<0.001** |
| Sex (Female) | -11066.564 (-12622.087 – -9511.040) | **<0.001** | -10800.560 (-12358.299 – -9242.821) | **<0.001** | -11042.504 (-12598.104 – -9486.904) | **<0.001** | -10955.281 (-12511.348 – -9399.214) | **<0.001** |
| Age | -728.104 (-823.634 – -632.573) | **<0.001** | -727.706 (-823.293 – -632.120) | **<0.001** | -716.931 (-812.247 – -621.615) | **<0.001** | -727.488 (-822.892 – -632.085) | **<0.001** |
| Intracranial Volume | 0.284 (0.278 – 0.289) | **<0.001** | 0.286 (0.281 – 0.291) | **<0.001** | 0.285 (0.279 – 0.290) | **<0.001** | 0.284 (0.279 – 0.290) | **<0.001** |
| R^2^ / R^2^ adjusted | 0.706 / 0.706 | | 0.704 / 0.704 | | 0.706 / 0.705 | | 0.705 / 0.705 | |

| **Table S7: Model Output for SES Predicting Composite Neurocognitive Score** | | | | | | | | |
| --- | --- | --- | --- | --- | --- | --- | --- | --- |
|  | **Neurocognition (Extended)** | | **Neurocognition (ADI)** | | **Neurocognition (PE)** | | **Neurocognition (HI)** | |
| *Predictors* | *Estimate (95% CI)* | *p* | *Estimate (95% CI)* | *p* | *Estimate (95% CI)* | *p* | *Estimate (95% CI)* | *p* |
| Intercepts | 57.340 (50.038 – 64.641) | **<0.001** | 69.523 (62.121 – 76.925) | **<0.001** | 50.016 (42.880 – 57.152) | **<0.001** | 56.149 (49.074 – 63.223) | **<0.001** |
| ADI | -0.055 (-0.071 – -0.039) | **<0.001** | -0.105 (-0.121 – -0.089) | **<0.001** |  |  |  |  |
| High School Grad/GED | 2.235 (0.221 – 4.249) | **0.092** |  |  | 2.461 (0.427 – 4.494) | **0.071** |  |  |
| Some College | 4.764 (2.869 – 6.658) | **<0.001** |  |  | 5.893 (3.997 – 7.789) | **<0.001** |  |  |
| Associate's/Bachelor's Degree | 7.678 (5.886 – 9.470) | **<0.001** |  |  | 10.375 (8.642 – 12.109) | **<0.001** |  |  |
| Postgraduate Degree | 11.991 (10.083 – 13.899) | **<0.001** |  |  | 16.132 (14.351 – 17.914) | **<0.001** |  |  |
| 50-<100K | 3.706 (2.648 – 4.764) | **<0.001** |  |  |  |  | 6.879 (5.877 – 7.881) | **<0.001** |
| >=100K | 4.803 (3.646 – 5.960) | **<0.001** |  |  |  |  | 10.470 (9.477 – 11.463) | **<0.001** |
| Physical Activity | 0.284 (0.128 – 0.440) | **0.001** |  |  |  |  |  |  |
| Race (Black) | -7.880 (-8.914 – -6.846) | **<0.001** | -11.759 (-12.763 – -10.754) | **<0.001** | -9.719 (-10.719 – -8.719) | **<0.001** | -9.117 (-10.158 – -8.076) | **<0.001** |
| Race (Asian) | 4.097 (2.601 – 5.592) | **<0.001** | 4.425 (2.875 – 5.976) | **<0.001** | 4.941 (3.448 – 6.435) | **<0.001** | 5.303 (3.794 – 6.811) | **<0.001** |
| Race (Other) | -2.348 (-4.024 – -0.672) | **0.048** | -5.791 (-7.497 – -4.084) | **<0.001** | -2.774 (-4.464 – -1.084) | **0.01** | -3.791 (-5.484 – -2.097) | **<0.001** |
| Ethnicity (Hispanic) | -1.243 (-2.240 – -0.246) | **0.019** | -4.633 (-5.620 – -3.647) | **<0.001** | -1.922 (-2.917 – -0.926) | **<0.001** | -2.410 (-3.410 – -1.410) | **<0.001** |
| Sex (Female) | 2.293 (1.507 – 3.079) | **<0.001** | 2.784 (1.970 – 3.597) | **<0.001** | 2.350 (1.557 – 3.144) | **<0.001** | 2.536 (1.735 – 3.337) | **<0.001** |
| Age | 0.165 (0.116 – 0.213) | **<0.001** | 0.159 (0.109 – 0.209) | **<0.001** | 0.181 (0.132 – 0.230) | **<0.001** | 0.162 (0.113 – 0.211) | **<0.001** |
| Intracranial Volume | 0.000 (0.000 – 0.000) | **<0.001** | 0.000 (0.000 – 0.000) | **<0.001** | 0.000 (0.000 – 0.000) | **<0.001** | 0.000 (0.000 – 0.000) | **<0.001** |
| R^2^ / R^2^ adjusted | 0.230 / 0.228 | | 0.170 / 0.169 | | 0.213 / 0.212 | | 0.196 / 0.195 | |

| **Table S8: Model Output for Physical Activity Predicting BMI** | | |
| --- | --- | --- |
|  | **BMI** | |
| *Predictors* | *Estimate (95% CI)* | *p* |
| Intercepts | 11.395 (9.760 – 13.029) | **<0.001** |
| Physical Activity | -0.084 (-0.120 – -0.047) | **<0.001** |
| Race (Black) | 1.955 (1.732 – 2.179) | **<0.001** |
| Race (Asian) | -0.372 (-0.720 – -0.023) | **0.037** |
| Race (Other) | 0.830 (0.442 – 1.218) | **<0.001** |
| Ethnicity (Hispanic) | 1.609 (1.384 – 1.834) | **<0.001** |
| Sex (Female) | 0.186 (0.001 – 0.372) | **0.048** |
| Age | 0.046 (0.035 – 0.058) | **<0.001** |
| Intracranial Volume | 0.000 (0.000 – 0.000) | **0.014** |
| R^2^ / R^2^ adjusted | 0.076 / 0.075 | |

| **Table S9: Model Output of Interaction Effects between BMI and Parental Education** | | | | |
| --- | --- | --- | --- | --- |
|  | **Total Volume** | | **Composite Neurocognitive Score** | |
| *Predictors* | *Estimate (95% CI)* | *p* | *Estimate (95% CI)* | *p* |
| Intercepts | 266111.37 (246567.75 – 285654.98) | **<0.001** | 49.17 (39.19 – 59.15) | **<0.001** |
| High School Grad/GED | -4152.81 (-22257.69 – 13952.08) | 0.653 | 4.98 (-4.26 – 14.22) | 0.653 |
| Some College | -13930.64 (-30814.86 – 2953.58) | 0.137 | 6.55 (-2.07 – 15.17) | 0.137 |
| Associate's/Bachelor's Degree | 1227.83 (-14105.58 – 16561.24) | 0.875 | 13.43 (5.60 – 21.26) | **0.002** |
| Postgraduate Degree | 6078.88 (-9678.72 – 21836.49) | 0.450 | 19.99 (11.95 – 28.04) | **<0.001** |
| BMI | -585.38 (-1257.26 – 86.50) | 0.176 | -0.04 (-0.38 – 0.31) | 0.834 |
| Race (Black) | -23595.66 (-25578.22 – -21613.10) | **<0.001** | -9.43 (-10.44 – -8.41) | **<0.001** |
| Race (Asian) | -3286.35 (-6210.46 – -362.25) | **0.028** | 4.90 (3.40 – 6.39) | **<0.001** |
| Race (Other) | 1359.97 (-1950.09 – 4670.03) | 0.421 | -2.70 (-4.39 – -1.01) | **0.004** |
| Ethnicity (Hispanic) | -4496.23 (-6461.87 – -2530.60) | **<0.001** | -1.68 (-2.68 – -0.67) | **0.001** |
| Sex (Female) | -10941.51 (-12495.64 – -9387.38) | **<0.001** | 2.40 (1.60 – 3.19) | **<0.001** |
| Age | -694.83 (-790.39 – -599.26) | **<0.001** | 0.19 (0.14 – 0.24) | **<0.001** |
| Intracranial Volume | 0.29 (0.28 – 0.29) | **<0.001** | 0.00 (0.00 – 0.00) | **<0.001** |
| High School Grad/GED : BMI | 43.83 (-817.63 – 905.29) | 0.921 | -0.12 (-0.56 – 0.32) | 0.921 |
| Some College : BMI | 745.26 (-66.56 – 1557.08) | 0.144 | -0.03 (-0.45 – 0.38) | 0.882 |
| Associate's/Bachelor's Degree : BMI | 10.22 (-726.30 – 746.74) | 0.978 | -0.16 (-0.54 – 0.21) | 0.796 |
| Postgraduate Degree : BMI | -34.79 (-803.54 – 733.96) | 0.929 | -0.21 (-0.61 – 0.18) | 0.574 |
| R^2^ / R^2^ adjusted | 0.707 / 0.706 | | 0.215 / 0.213 | |

| **Table S10: Model Output of Interaction Effects between BMI and Household Income** | | | | |
| --- | --- | --- | --- | --- |
|  | **Total Volume** | | **Composite Neurocognitive Score** | |
| *Predictors* | *Estimate (95% CI)* | *p* | *Estimate (95% CI)* | *p* |
| Intercepts | 263160.57 (248377.92 – 277943.21) | **<0.001** | 55.34 (47.72 – 62.95) | **<0.001** |
| 50-<100K | 1583.10 (-7309.91 – 10476.10) | 0.727 | 11.98 (7.40 – 16.56) | **<0.001** |
| >=100K | 10808.57 (2175.57 – 19441.57) | **0.014** | 14.53 (10.08 – 18.98) | **<0.001** |
| BMI | -414.66 (-712.69 – -116.64) | **0.012** | -0.06 (-0.22 – 0.09) | 0.409 |
| Race (Black) | -23282.91 (-25322.67 – -21243.15) | **<0.001** | -8.75 (-9.80 – -7.70) | **<0.001** |
| Race (Asian) | -3096.04 (-6021.26 – -170.82) | **0.038** | 5.25 (3.74 – 6.76) | **<0.001** |
| Race (Other) | 1197.31 (-2088.81 – 4483.42) | 0.475 | -3.63 (-5.32 – -1.94) | **<0.001** |
| Ethnicity (Hispanic) | -4471.39 (-6427.89 – -2514.89) | **<0.001** | -2.09 (-3.10 – -1.08) | **<0.001** |
| Sex (Female) | -10829.93 (-12384.54 – -9275.32) | **<0.001** | 2.57 (1.77 – 3.38) | **<0.001** |
| Age | -705.32 (-800.99 – -609.66) | **<0.001** | 0.17 (0.12 – 0.22) | **<0.001** |
| Intracranial Volume | 0.29 (0.28 – 0.29) | **<0.001** | 0.00 (0.00 – 0.00) | **<0.001** |
| 50-<100K : BMI | 89.18 (-362.22 – 540.58) | 0.699 | -0.27 (-0.50 – -0.04) | **0.044** |
| >=100K : BMI | -278.11 (-727.99 – 171.77) | 0.226 | -0.23 (-0.46 – 0.01) | 0.112 |
| R^2^ / R^2^ adjusted | 0.706 / 0.706 | | 0.199 / 0.198 | |

| **Table S11: Model Output for SES Predicting Prefrontal Cortical Volume** | | | | | | | | |
| --- | --- | --- | --- | --- | --- | --- | --- | --- |
|  | **Prefrontal Volume (Extended)** | | **Prefrontal Volume (ADI)** | | **Prefrontal Volume (PE)** | | **Prefrontal Volume (HI)** | |
| *Predictors* | *Estimate (95% CI)* | *p* | *Estimate (95% CI)* | *p* | *Estimate (95% CI)* | *p* | *Estimate (95% CI)* | *p* |
| Intercepts | 79420.806 (74598.031 – 84243.581) | **<0.001** | 80932.322 (76207.060 – 85657.584) | **<0.001** | 78580.897 (73913.254 – 83248.540) | **<0.001** | 78990.879 (74408.102 – 83573.656) | **<0.001** |
| ADI | -5.546 (-16.006 – 4.914) | 0.341 | -15.345 (-25.292 – -5.397) | **0.003** |  |  |  |  |
| High School Grad/GED | -743.123 (-2073.533 – 587.287) | 0.438 |  |  | -696.907 (-2026.763 – 632.948) | 0.487 |  |  |
| Some College | 446.274 (-805.051 – 1697.599) | 0.554 |  |  | 638.827 (-601.524 – 1879.178) | 0.417 |  |  |
| Associate's/Bachelor's Degree | 361.163 (-822.542 – 1544.867) | 0.733 |  |  | 837.640 (-296.304 – 1971.583) | 0.197 |  |  |
| Postgraduate Degree | 1443.245 (182.821 – 2703.669) | **0.033** |  |  | 2190.378 (1024.950 – 3355.806) | **<0.001** |  |  |
| 50-<100K | 618.179 (-80.467 – 1316.824) | 0.095 |  |  |  |  | 1073.816 (424.795 – 1722.836) | **0.001** |
| >=100K | 982.989 (218.790 – 1747.189) | **0.013** |  |  |  |  | 1859.162 (1216.010 – 2502.313) | **<0.001** |
| Physical Activity | 59.816 (-43.124 – 162.757) | 0.340 |  |  |  |  |  |  |
| Race (Black) | -4406.211 (-5089.264 – -3723.158) | **<0.001** | -5111.966 (-5753.071 – -4470.862) | **<0.001** | -4716.287 (-5370.529 – -4062.044) | **<0.001** | -4605.350 (-5279.564 – -3931.137) | **<0.001** |
| Race (Asian) | -1779.694 (-2767.760 – -791.628) | **0.001** | -1715.591 (-2705.328 – -725.855) | **0.001** | -1686.578 (-2663.396 – -709.760) | **0.001** | -1617.528 (-2594.588 – -640.467) | **0.002** |
| Race (Other) | 246.050 (-860.807 – 1352.908) | 0.663 | -281.269 (-1370.503 – 807.965) | 0.700 | 163.735 (-941.552 – 1269.022) | 0.772 | 70.256 (-1026.739 – 1167.250) | 0.900 |
| Ethnicity (Hispanic) | -2003.358 (-2662.069 – -1344.648) | **<0.001** | -2556.883 (-3186.459 – -1927.306) | **<0.001** | -2146.211 (-2797.418 – -1495.003) | **<0.001** | -2147.414 (-2795.235 – -1499.594) | **<0.001** |
| Sex (Female) | -3152.546 (-3671.606 – -2633.486) | **<0.001** | -3070.869 (-3590.268 – -2551.471) | **<0.001** | -3145.793 (-3664.701 – -2626.884) | **<0.001** | -3117.719 (-3636.749 – -2598.690) | **<0.001** |
| Age | -182.441 (-214.318 – -150.563) | **<0.001** | -182.578 (-214.450 – -150.707) | **<0.001** | -179.477 (-211.272 – -147.682) | **<0.001** | -182.571 (-214.392 – -150.749) | **<0.001** |
| Intracranial Volume | 0.074 (0.072 – 0.075) | **<0.001** | 0.074 (0.073 – 0.076) | **<0.001** | 0.074 (0.072 – 0.076) | **<0.001** | 0.074 (0.072 – 0.076) | **<0.001** |
| R^2^ / R^2^ adjusted | 0.591 / 0.591 | | 0.589 / 0.588 | | 0.591 / 0.590 | | 0.590 / 0.590 | |

| **Table S12: Model Output for SES Predicting Occipital Cortical Volume** | | | | | | | | |
| --- | --- | --- | --- | --- | --- | --- | --- | --- |
|  | **Occipital Volume (Extended)** | | **Occipital Volume (ADI)** | | **Occipital Volume (PE)** | | **Occipital Volume (HI)** | |
| *Predictors* | *Estimate (95% CI)* | *p* | *Estimate (95% CI)* | *p* | *Estimate (95% CI)* | *p* | *Estimate (95% CI)* | *p* |
| Intercepts | 27242.213 (25042.927 – 29441.498) | **<0.001** | 27500.917 (25349.508 – 29652.326) | **<0.001** | 27159.103 (25031.383 – 29286.823) | **<0.001** | 27088.132 (25000.103 – 29176.161) | **<0.001** |
| ADI | -0.209 (-4.979 – 4.561) | 0.932 | -2.919 (-7.448 – 1.610) | 0.206 |  |  |  |  |
| High School Grad/GED | -654.004 (-1260.699 – -47.310) | **0.092** |  |  | -634.150 (-1240.357 – -27.942) | 0.108 |  |  |
| Some College | -221.830 (-792.460 – 348.800) | 0.554 |  |  | -153.890 (-719.297 – 411.517) | 0.594 |  |  |
| Associate's/Bachelor's Degree | 85.369 (-454.425 – 625.163) | 0.757 |  |  | 244.590 (-272.312 – 761.492) | 0.354 |  |  |
| Postgraduate Degree | 75.838 (-498.941 – 650.618) | 0.796 |  |  | 313.209 (-218.045 – 844.463) | 0.248 |  |  |
| 50-<100K | 237.446 (-81.151 – 556.043) | 0.144 |  |  |  |  | 384.610 (88.900 – 680.320) | **0.011** |
| >=100K | 350.735 (2.244 – 699.226) | **0.049** |  |  |  |  | 549.833 (256.796 – 842.869) | **<0.001** |
| Physical Activity | 14.619 (-32.324 – 61.562) | 0.542 |  |  |  |  |  |  |
| Race (Black) | -4084.665 (-4396.151 – -3773.179) | **<0.001** | -4332.427 (-4624.322 – -4040.532) | **<0.001** | -4176.422 (-4474.655 – -3878.189) | **<0.001** | -4155.097 (-4462.286 – -3847.909) | **<0.001** |
| Race (Asian) | -673.400 (-1123.979 – -222.822) | **0.005** | -664.207 (-1114.834 – -213.581) | **0.006** | -664.555 (-1109.832 – -219.278) | **0.006** | -658.539 (-1103.713 – -213.366) | **0.006** |
| Race (Other) | -135.512 (-640.262 – 369.239) | 0.663 | -296.048 (-791.975 – 199.880) | 0.323 | -165.843 (-669.682 – 337.997) | 0.593 | -181.698 (-681.516 – 318.121) | 0.635 |
| Ethnicity (Hispanic) | -478.803 (-779.189 – -178.417) | **0.004** | -630.022 (-916.667 – -343.376) | **<0.001** | -526.081 (-822.930 – -229.232) | **0.001** | -504.658 (-799.822 – -209.495) | **0.001** |
| Sex (Female) | -1602.616 (-1839.318 – -1365.914) | **<0.001** | -1579.346 (-1815.828 – -1342.864) | **<0.001** | -1600.183 (-1836.724 – -1363.641) | **<0.001** | -1595.804 (-1832.287 – -1359.322) | **<0.001** |
| Age | -82.110 (-96.646 – -67.573) | **<0.001** | -81.953 (-96.464 – -67.442) | **<0.001** | -81.395 (-95.889 – -66.901) | **<0.001** | -82.017 (-96.516 – -67.518) | **<0.001** |
| Intracranial Volume | 0.026 (0.025 – 0.027) | **<0.001** | 0.026 (0.025 – 0.027) | **<0.001** | 0.026 (0.025 – 0.027) | **<0.001** | 0.026 (0.025 – 0.027) | **<0.001** |
| R^2^ / R^2^ adjusted | 0.513 / 0.512 | | 0.511 / 0.511 | | 0.513 / 0.512 | | 0.512 / 0.512 | |

| **Table S13: Model Output for SES Predicting Flanker Task Performance** | | | | | | | | |
| --- | --- | --- | --- | --- | --- | --- | --- | --- |
|  | **Flanker (Extended)** | | **Flanker (ADI)** | | **Flanker (PE)** | | **Flanker (HI)** | |
| *Predictors* | *Estimate (95% CI)* | *p* | *Estimate (95% CI)* | *p* | *Estimate (95% CI)* | *p* | *Estimate (95% CI)* | *p* |
| Intercepts | 83.878 (77.637 – 90.118) | **<0.001** | 87.586 (81.465 – 93.708) | **<0.001** | 80.329 (74.276 – 86.382) | **<0.001** | 82.414 (76.480 – 88.348) | **<0.001** |
| ADI | -0.027 (-0.041 – -0.014) | **<0.001** | -0.041 (-0.054 – -0.028) | **<0.001** |  |  |  |  |
| High School Grad/GED | 0.549 (-1.173 – 2.270) | 0.608 |  |  | 0.642 (-1.082 – 2.367) | 0.532 |  |  |
| Some College | 2.374 (0.755 – 3.994) | **0.011** |  |  | 2.872 (1.263 – 4.481) | **0.001** |  |  |
| Associate's/Bachelor's Degree | 2.419 (0.887 – 3.951) | **0.004** |  |  | 3.614 (2.144 – 5.085) | **<0.001** |  |  |
| Postgraduate Degree | 2.793 (1.162 – 4.424) | **0.001** |  |  | 4.624 (3.113 – 6.136) | **<0.001** |  |  |
| 50-<100K | 1.678 (0.774 – 2.582) | **0.001** |  |  |  |  | 2.460 (1.620 – 3.300) | **<0.001** |
| >=100K | 2.152 (1.163 – 3.141) | **<0.001** |  |  |  |  | 3.477 (2.644 – 4.310) | **<0.001** |
| Physical Activity | 0.048 (-0.085 – 0.181) | 0.542 |  |  |  |  |  |  |
| Race (Black) | -1.486 (-2.370 – -0.602) | **0.001** | -2.644 (-3.475 – -1.814) | **<0.001** | -2.315 (-3.163 – -1.466) | **<0.001** | -1.838 (-2.711 – -0.965) | **<0.001** |
| Race (Asian) | 3.722 (2.443 – 5.000) | **<0.001** | 3.783 (2.500 – 5.065) | **<0.001** | 4.157 (2.890 – 5.424) | **<0.001** | 4.172 (2.907 – 5.438) | **<0.001** |
| Race (Other) | -1.000 (-2.432 – 0.432) | 0.456 | -2.056 (-3.467 – -0.645) | **0.009** | -1.184 (-2.617 – 0.250) | 0.281 | -1.393 (-2.813 – 0.028) | 0.109 |
| Ethnicity (Hispanic) | 1.078 (0.225 – 1.930) | **0.019** | 0.096 (-0.719 – 0.912) | 0.817 | 0.801 (-0.044 – 1.645) | 0.063 | 0.811 (-0.028 – 1.650) | 0.058 |
| Sex (Female) | -0.396 (-1.068 – 0.276) | 0.283 | -0.261 (-0.934 – 0.411) | 0.446 | -0.357 (-1.030 – 0.316) | 0.323 | -0.338 (-1.010 – 0.334) | 0.324 |
| Age | 0.050 (0.009 – 0.092) | **0.017** | 0.050 (0.009 – 0.091) | **0.018** | 0.056 (0.015 – 0.098) | **0.007** | 0.052 (0.010 – 0.093) | **0.014** |
| Intracranial Volume | 0.000 (0.000 – 0.000) | **0.01** | 0.000 (0.000 – 0.000) | **<0.001** | 0.000 (0.000 – 0.000) | **0.002** | 0.000 (0.000 – 0.000) | **0.004** |
| R^2^ / R^2^ adjusted | 0.036 / 0.035 | | 0.028 / 0.027 | | 0.031 / 0.030 | | 0.032 / 0.031 | |

| **Table S14: Model Output for SES Predicting List Sort Task Performance** | | | | | | | | |
| --- | --- | --- | --- | --- | --- | --- | --- | --- |
|  | **List Sort (Extended)** | | **List Sort (ADI)** | | **List Sort (PE)** | | **List Sort (HI)** | |
| *Predictors* | *Estimate (95% CI)* | *p* | *Estimate (95% CI)* | *p* | *Estimate (95% CI)* | *p* | *Estimate (95% CI)* | *p* |
| Intercepts | 77.248 (70.816 – 83.679) | **<0.001** | 85.265 (78.863 – 91.667) | **<0.001** | 73.047 (66.790 – 79.303) | **<0.001** | 77.406 (71.241 – 83.572) | **<0.001** |
| ADI | -0.031 (-0.045 – -0.017) | **<0.001** | -0.062 (-0.075 – -0.048) | **<0.001** |  |  |  |  |
| High School Grad/GED | 2.025 (0.251 – 3.799) | 0.092 |  |  | 2.175 (0.393 – 3.958) | **0.071** |  |  |
| Some College | 4.052 (2.383 – 5.720) | **<0.001** |  |  | 4.761 (3.099 – 6.424) | **<0.001** |  |  |
| Associate's/Bachelor's Degree | 5.197 (3.619 – 6.776) | **<0.001** |  |  | 6.907 (5.387 – 8.427) | **<0.001** |  |  |
| Postgraduate Degree | 7.708 (6.027 – 9.389) | **<0.001** |  |  | 10.367 (8.805 – 11.929) | **<0.001** |  |  |
| 50-<100K | 2.235 (1.303 – 3.167) | **<0.001** |  |  |  |  | 4.158 (3.285 – 5.031) | **<0.001** |
| >=100K | 3.080 (2.061 – 4.099) | **<0.001** |  |  |  |  | 6.414 (5.548 – 7.279) | **<0.001** |
| Physical Activity | 0.295 (0.158 – 0.432) | **<0.001** |  |  |  |  |  |  |
| Race (Black) | -4.388 (-5.299 – -3.477) | **<0.001** | -6.747 (-7.615 – -5.878) | **<0.001** | -5.541 (-6.418 – -4.664) | **<0.001** | -5.093 (-6.000 – -4.186) | **<0.001** |
| Race (Asian) | 0.787 (-0.531 – 2.104) | 0.277 | 0.943 (-0.398 – 2.284) | 0.192 | 1.234 (-0.075 – 2.544) | 0.074 | 1.435 (0.120 – 2.749) | **0.043** |
| Race (Other) | -0.755 (-2.232 – 0.721) | 0.631 | -2.931 (-4.407 – -1.455) | **<0.001** | -1.034 (-2.515 – 0.448) | 0.343 | -1.703 (-3.179 – -0.227) | **0.063** |
| Ethnicity (Hispanic) | -0.684 (-1.563 – 0.194) | 0.145 | -2.849 (-3.702 – -1.996) | **<0.001** | -1.162 (-2.035 – -0.290) | **0.012** | -1.477 (-2.348 – -0.605) | **0.001** |
| Sex (Female) | 0.335 (-0.357 – 1.027) | 0.342 | 0.615 (-0.089 – 1.318) | 0.099 | 0.351 (-0.345 – 1.046) | 0.323 | 0.460 (-0.238 – 1.158) | 0.225 |
| Age | 0.067 (0.024 – 0.109) | **0.002** | 0.066 (0.023 – 0.109) | **0.003** | 0.079 (0.037 – 0.122) | **<0.001** | 0.068 (0.025 – 0.110) | **0.002** |
| Intracranial Volume | 0.000 (0.000 – 0.000) | **<0.001** | 0.000 (0.000 – 0.000) | **<0.001** | 0.000 (0.000 – 0.000) | **<0.001** | 0.000 (0.000 – 0.000) | **<0.001** |
| R^2^ / R^2^ adjusted | 0.116 / 0.114 | | 0.081 / 0.080 | | 0.105 / 0.104 | | 0.097 / 0.096 | |

| **Table S15: Model Output for SES Predicting Card Sort Task Performance** | | | | | | | | |
| --- | --- | --- | --- | --- | --- | --- | --- | --- |
|  | **Card Sort (Extended)** | | **Card Sort (ADI)** | | **Card Sort (PE)** | | **Card Sort (HI)** | |
| *Predictors* | *Estimate (95% CI)* | *p* | *Estimate (95% CI)* | *p* | *Estimate (95% CI)* | *p* | *Estimate (95% CI)* | *p* |
| Intercepts | 81.326 (74.433 – 88.219) | **<0.001** | 85.748 (78.971 – 92.526) | **<0.001** | 78.120 (71.436 – 84.804) | **<0.001** | 80.089 (73.527 – 86.651) | **<0.001** |
| ADI | -0.024 (-0.039 – -0.009) | **0.002** | -0.044 (-0.058 – -0.030) | **<0.001** |  |  |  |  |
| High School Grad/GED | 0.680 (-1.221 – 2.582) | 0.608 |  |  | 0.802 (-1.103 – 2.706) | 0.532 |  |  |
| Some College | 1.290 (-0.499 – 3.078) | 0.252 |  |  | 1.860 (0.084 – 3.636) | **0.064** |  |  |
| Associate's/Bachelor's Degree | 2.320 (0.628 – 4.012) | **0.012** |  |  | 3.621 (1.997 – 5.245) | **<0.001** |  |  |
| Postgraduate Degree | 4.010 (2.209 – 5.812) | **<0.001** |  |  | 5.940 (4.271 – 7.608) | **<0.001** |  |  |
| 50-<100K | 1.952 (0.953 – 2.950) | **<0.001** |  |  |  |  | 3.056 (2.127 – 3.986) | **<0.001** |
| >=100K | 2.221 (1.129 – 3.314) | **<0.001** |  |  |  |  | 4.316 (3.395 – 5.237) | **<0.001** |
| Physical Activity | 0.133 (-0.015 – 0.280) | 0.155 |  |  |  |  |  |  |
| Race (Black) | -3.536 (-4.512 – -2.559) | **<0.001** | -5.107 (-6.027 – -4.188) | **<0.001** | -4.388 (-5.325 – -3.451) | **<0.001** | -4.012 (-4.977 – -3.047) | **<0.001** |
| Race (Asian) | 2.336 (0.924 – 3.748) | **0.002** | 2.450 (1.031 – 3.870) | **0.001** | 2.703 (1.304 – 4.102) | **<0.001** | 2.829 (1.430 – 4.228) | **<0.001** |
| Race (Other) | -0.574 (-2.156 – 1.008) | 0.636 | -1.878 (-3.440 – -0.315) | **0.030** | -0.784 (-2.366 – 0.799) | 0.531 | -1.036 (-2.606 – 0.535) | 0.314 |
| Ethnicity (Hispanic) | -0.647 (-1.589 – 0.294) | 0.178 | -1.947 (-2.850 – -1.044) | **<0.001** | -0.951 (-1.884 – -0.019) | **0.052** | -1.037 (-1.964 – -0.109) | **0.033** |
| Sex (Female) | 1.921 (1.179 – 2.662) | **<0.001** | 2.107 (1.362 – 2.852) | **<0.001** | 1.948 (1.205 – 2.691) | **<0.001** | 2.003 (1.260 – 2.747) | **<0.001** |
| Age | 0.083 (0.037 – 0.129) | **<0.001** | 0.082 (0.036 – 0.127) | **0.001** | 0.090 (0.045 – 0.136) | **<0.001** | 0.083 (0.037 – 0.129) | **<0.001** |
| Intracranial Volume | 0.000 (-0.000 – 0.000) | 0.071 | 0.000 (0.000 – 0.000) | **0.002** | 0.000 (0.000 – 0.000) | **0.021** | 0.000 (0.000 – 0.000) | **0.023** |
| R^2^ / R^2^ adjusted | 0.054 / 0.052 | | 0.042 / 0.041 | | 0.050 / 0.048 | | 0.048 / 0.047 | |

**Table S16: Mediation Analysis**

| *Measures* | *Indirect Effect* | *CI* | *p* | *Proportion Mediated* | *p* |
| --- | --- | --- | --- | --- | --- |
| Total Cortical Volume | -7.01 | -10.49 - -4.16 | **<0 .001** | 0.1225 | **<0.001** |
| Prefrontal Cortical Volume | -2.58 | -3.73 - -1.56 | **<0 .001** | 0.1692 | **<0.001** |
| Composite Neurocognitive Score | -.0039 | -0.0057 –  -0.0027 | **<0.001** | 0.0368 | **<0.001** |
| Flanker Task | -0.00079 | -0.0019 - 0.0002 | 0.17 | 0.0199 | 0.17 |
| List Sort Task | -0.0026 | -0.0039 -  - 0.0014 | **<0.001** | 0.0414 | **<0.001** |
| Card Sort Task | -0.0025 | -0.0040 –  -0.0013 | **<0.001** | 0.0565 | **<0.001** |

**Quadratic Models**

In addition to our extended multiple linear regression models of BMI, Total Cortical Volume, and Composite Neurocognitive Score, we also included a set of analyses with a quadratic term for Area Deprivation Index, as an exploratory analysis to investigate if a polynomial regression would more optimally fit the data. The quadratic term was significant in the models predicting BMI (𝜷 = 0.0002, P < 0.001) and neurocognition (𝜷 = -0.001, P < 0.001), but not cortical volume (𝜷 = 0.09, P =.8219). Though the quadratic term is significantly related to BMI and neurocognition, we report these analyses for completeness and do not attempt to interpret the quadratic relationship between ADI and our outcome variables, since we had no *a priori* hypothesis regarding polynomial trends. Nevertheless, complex fits, like the quadratic regression may be useful when the primary goal is prediction rather than hypothesis testing as is the case in the current study.

| **Table S17: Models with Quadratic ADI Term** | | | | | | |
| --- | --- | --- | --- | --- | --- | --- |
|  | **BMI** | | **Cortical Volume** | | **Neurocognition** | |
| *Predictors* | *Estimate (95% CI)* | *p* | *Estimate (95% CI)* | *p* | *Estimate (95% CI)* | *p* |
| Intercepts | 11.698 (9.992 – 13.404) | **<0.001** | 261959.106 (247444.217 – 276473.995) | **<0.001** | 55.790 (48.467 – 63.113) | **<0.001** |
| ADI | -0.022 (-0.035 – -0.009) | **0.001** | -35.814 (-145.074 – 73.445) | 0.521 | 0.067 (0.012 – 0.122) | **0.018** |
| ADI^2 | 0.0002 (0.000 – 0.000) | **<0.001** | 0.093 (-0.714 – 0.900) | 0.822 | -0.001 (-0.001 – -0.001) | **<0.001** |
| High School Grad/GED | -0.093 (-0.562 – 0.376) | 0.697 | -3355.719 (-7343.306 – 631.869) | 0.099 | 2.297 (0.285 – 4.308) | **0.025** |
| Some College | -0.486 (-0.927 – -0.046) | **0.031** | 416.924 (-3333.740 – 4167.588) | 0.828 | 4.696 (2.804 – 6.589) | **<0.001** |
| Associate's/Bachelor's Degree | -1.028 (-1.445 – -0.611) | **<0.001** | 630.858 (-2918.726 – 4180.442) | 0.728 | 7.539 (5.748 – 9.329) | **<0.001** |
| Postgraduate Degree | -1.298 (-1.743 – -0.854) | **<0.001** | 4007.770 (224.051 – 7791.489) | **0.038** | 11.738 (9.829 – 13.647) | **<0.001** |
| 50-<100K | -0.251 (-0.499 – -0.004) | **0.047** | 2042.062 (-65.651 – 4149.774) | 0.058 | 3.425 (2.361 – 4.488) | **<0.001** |
| >=100K | -0.540 (-0.815 – -0.266) | **<0.001** | 3487.694 (1153.184 – 5822.203) | **0.003** | 4.276 (3.098 – 5.454) | **<0.001** |
| Physical Activity | -0.052 (-0.088 – -0.015) | **0.005** | 218.560 (-90.095 – 527.214) | 0.165 | 0.273 (0.117 – 0.429) | **0.001** |
| Race (Black) | 1.188 (0.943 – 1.432) | **<0.001** | -23252.011 (-25335.672 – -21168.351) | **<0.001** | -7.427 (-8.479 – -6.376) | **<0.001** |
| Race (Asian) | -0.092 (-0.440 – 0.257) | 0.606 | -3556.595 (-6518.707 – -594.482) | **0.019** | 4.012 (2.518 – 5.507) | **<0.001** |
| Race (Other) | 0.403 (0.013 – 0.793) | **0.043** | 1442.639 (-1876.318 – 4761.597) | 0.394 | -2.472 (-4.146 – -0.797) | **0.004** |
| Ethnicity (Hispanic) | 1.200 (0.968 – 1.433) | **<0.001** | -4639.311 (-6618.771 – -2659.851) | **<0.001** | -1.412 (-2.410 – -0.413) | **0.006** |
| Sex (Female) | 0.274 (0.091 – 0.457) | **0.003** | -11065.286 (-12620.947 – -9509.626) | **<0.001** | 2.280 (1.495 – 3.065) | **<0.001** |
| Age | 0.046 (0.035 – 0.058) | **<0.001** | -727.903 (-823.455 – -632.351) | **<0.001** | 0.162 (0.114 – 0.211) | **<0.001** |
| Intracranial Volume | 0.000 (0.000 – 0.000) | **<0.001** | 0.284 (0.278 – 0.289) | **<0.001** | 0.000 (0.000 – 0.000) | **<0.001** |
| R^2^ / R^2^ adjusted | 0.104 / 0.102 | | 0.706 / 0.706 | | 0.232 / 0.230 | |
